# Clinical Analysis of the Discovery of Malignant Gynecological Tumors in the Diagnosis and Treatment of Pelvic Organ Prolapse

**DOI:** 10.3389/fsurg.2022.877857

**Published:** 2022-05-16

**Authors:** Li-Na Niu, Jun-Xia Wang, Xia Li, Yong-Jun Xu, Li-Rong Qiu, Sheng Guo, Li-Zhen Zhang, Yun Shang

**Affiliations:** ^1^ Department of Gynecology, Yuncheng Central Hospital, Yuncheng, China; ^2^ Department of General Family medicine, Yuncheng Central Hospital, Yuncheng, China; ^3^ Department of Pharmacy, Yuncheng Central Hospital, Yuncheng, China

**Keywords:** pelvic organ prolapse, gynecological malignancies, laparoscope, total pelvic reconstruction without mesh, diagnosis, treatment

## Abstract

**Background:**

Clinically, malignant gynecological tumors found by chance during the diagnosis and treatment of pelvic organ prolapse (POP) are rare, and they are usually missed, leading to delayed diagnosis and treatment. The initial treatment of these tumors cannot be standardized, and, as a single surgical intervention may not be able to treat both the tumor and prolapse, secondary surgery is usually needed, affecting the quality of life of patients.

**Case presentation:**

The present study retrospectively analyzed the data of three patients who were diagnosed with malignant gynecological tumors during the diagnosis and treatment of POP. These patients were among 215 patients with POP treated in Yuncheng Central Hospital of Shanxi Province between January 2011 and May 2020. The case characteristics, surgical interventions, postoperative treatments, and follow-ups were summarized, and the characteristics of diagnosis and treatment were analyzed in the context of relevant literature.

**Conclusion:**

As long as clinicians operate in strict accordance with the standards of diagnosis and treatment, obtain a complete medical history, undertake a physical examination, and remain diligent in auxiliary examinations, following existing clinical methods and diagnosis and treatment processes, patients with POP complicated with malignant gynecological tumors can be clearly diagnosed before and during surgery. In this way, initial treatment can be standardized, and surgical methods can be selected that address both the tumor and prolapse, thereby avoiding secondary surgery and improving the patient’s quality of life.

## Introduction

Pelvic organ prolapse (POP) is a common benign disease in middle-aged and elderly women, with its incidence increasing with age (1). The etiology of the disease is unclear, and there are many disputes about its diagnosis, treatment, and appropriate surgical methods. Furthermore, there is no unified standard for its diagnosis and treatment, and the preferences of patients may differ: for example, previous surveys have found that 36% of women with POP wish to retain their uterus during the correction of POP (2, 3).

There are many categories of malignant tumor that affect the female reproductive organs, with some being occult and easy to miss in clinic. However, their prognosis is closely related to tumor differentiation, stage, and treatment. Some malignant tumors can be removed in a single operation in the early stage (4). Therefore, the early detection and treatment of malignant gynecological tumors is vital. If preoperative evaluation ignores other possible problems in the diagnosis and treatment of POP, however, the patient’s treatment will be delayed, resulting in lost treatment opportunities and increasing the risk of secondary surgery. Avoiding this requires clinicians to fully evaluate patients with POP prior to surgery and identify and address any abnormalities. When formulating treatment strategies for patients with POP complicated with malignant gynecological tumors before surgery, clinicians should consider both the prolapse and the tumor.

In the present study, the general situations, diagnosis and treatment processes, and postoperative situations of three patients with POP complicated with malignant gynecological tumors were retrospectively analyzed. The analysis was then discussed in the context of relevant literature in order to identify ways of avoiding missed diagnosis of malignant gynecological tumors in these patients and explore the feasibility of laparoscopic surgery and total pelvic reconstruction without mesh in the treatment of this condition.

## Clinical Data

The present study retrospectively analyzed the data of three patients (1.39%) who were diagnosed with malignant gynecological tumors in the diagnosis and treatment of 215 patients with POP at Yuncheng Central Hospital of Shanxi Province between January 2011 and May 2020. The case characteristics, surgical interventions, postoperative treatments, and follow-ups were summarized, and the characteristics of diagnosis and treatment were analyzed in the context of relevant literature.

**Case 1:** A 62-year-old-patient who had been in natural menopause for 15 years, with no contact bleeding or vaginal discharge, was hospitalized on July 16, 2018, for prolapse surgery due to a uterine prolapse of nine months. After admission, a routine ThinPrep cytological test showed atypical squamous epithelial cells (high intraepithelial lesions could not be excluded). A colposcopy and cervical biopsy were performed. The pathological results indicated high-grade squamous intraepithelial lesions involving the glands. A loop electrical excision procedure (LEEP) was performed, and the pathology of the resected samples showed slight infiltration at the positions of 1, 2, 7, 10, and 11 o’clock, with depths of approximately 0.15 cm. Diagnosis was stage IA1 of cervical squamous cell carcinoma, grade III uterine prolapse, and grade I anterior vaginal wall prolapse. Laparoscopic extrafascial double adnexectomy and total pelvic reconstruction without mesh were performed on July 24, 2018. The operation was completed with no complications, and the patient was discharged three days later. No supplementary treatment was given. There has since been no recurrence of the tumor or prolapse, and the patient has experienced no discomfort. The details are presented in **Table 1**.

**Table 1:** The general conditions and operation conditions of the three patients.

Nomber	Age	Menopause	Symptoms other than prolapse	Main diagnose	Operation situations	Presence complications	Postoperative pathology	Postoperative treatment	Follow up (months)
Case 1	62	Yes	None	Stage IA1 cervical cancer Pelvic organ prolapse	Laparoscopic extrafascial double adnexectomy + total pelvic reconstruction without mesh	None	Cervical carcinoma	None	41
Case 2	50	Yes	Vaginal bleeding and drainage	Stage IA G3 serous carcinoma of fallopian tube Pelvic organ prolapse	Laparoscopic double adnexectomy and greater omentum and appendix resection + pelvic lymph node dissection + total pelvic reconstruction without mesh	None	Fallopian tube carcinoma	chemotherapy	21
Case 3	62	Yes	None	Stage IB G1 endometrial carcinoma Pelvic organ prolapse	laparoscopic double adnexectomy + pelvic lymph node dissection + total pelvic reconstruction without mesh	None	Endometrial carcinoma	Radiotherapy	21

**Case 2:** A 50-year-old patient who had been in natural menopause for eight years, with a previous history of breast cancer, was hospitalized on March 23, 2020, due to a uterine prolapse. The patient had experienced three instances of postmenopausal vaginal bleeding and had lower abdominal distension and discomfort with vaginal drainage for one month. A solid mass in the adnexal area (from the fallopian tube) was identified during the admission examination, and tumor markers were normal. Preoperative diagnosis: the nature of the pelvic mass remained to be checked but was suspected to be fallopian tube cancer, and a grade III uterine prolapse and grade II anterior vaginal wall prolapse were confirmed. The patient was fully communicated with before surgery, which was performed on March 27, 2020. Intraoperative frozen sections identified a malignant tumor in the left fallopian tube. Total uterus resection, double adnexectomy, omentum resection, vermiform appendix resection, pelvic lymph node dissection, and total pelvic reconstruction without mesh were performed. The operation was completed with no complications, and the patient was discharged six days later. Postoperative pathology indicated high-grade serous carcinoma of the left fallopian tube. Docetaxel and cyclophosphamide chemotherapy was given. There has since been no recurrence of the tumor or prolapse, and the patient has experienced no discomfort. The details are presented in **Table 1**.

**Case 3:** A 62-year-old patient who had been in natural menopause for 12 years, with no vaginal bleeding or drainage after menopause, was hospitalized due to a self-palpable vaginal mass that had been prolapsed for four months and aggravated for two months. Preoperative ultrasonography revealed that the thickness of the endometrium was 0.6 cm. A hysteroscopy was carried out, during which a polypoid lesion of approximately 1.0 cm could be seen in the left uterine horn. Electrosurgical examination revealed endometrial complex atypical hyperplasia and focal canceration. Preoperative diagnosis: endometrial cancer, grade III prolapse of the anterior vaginal wall, grade II prolapse of the posterior vaginal wall, type 2 diabetes mellitus, and grade I hypertension. On April 1, 2020, a laparoscopic extrafascial double adnexectomy, pelvic lymph node dissection, and total pelvic reconstruction without mesh were performed. The operation was completed with no complications, and the patient was discharged seven days later. Postoperative pathological examination revealed endometrial cancer with infiltration into the deep myometrium. The patient was treated with supplementary vaginal brachytherapy after operation. There has since been no recurrence of the tumor or prolapse, and the patient has experienced no discomfort. The details are presented in **Table 1**.

Of these patients, two had no clinical symptoms of malignant gynecological tumors. Abnormalities were found during routine examinations prior to surgery for POP, and all tumors were confirmed before and during surgery. The operation in Case 1 was a standardized operation, in which malignant gynecological tumor surgery and POP surgery were performed simultaneously. Total pelvic reconstruction without mesh was selected to treat the POP in all cases, and the surgical procedures were smooth and without complication. Secondary surgery was avoided, the quality of life of the patients was improved, and the complications related to the use of mesh were avoided.

## Discussion

Although the malignant gynecological tumors in all three patients were found by chance during the diagnosis and treatment of POP, the discoveries occurred during an early stage, the initial treatments were standardized, the corresponding supplementary treatments were given, and the prognoses were good. However, analysis and summary of these three cases highlighted the need to investigate the application of laparoscopic total pelvic reconstruction without mesh in patients with gynecological malignant tumors.

### Diagnosis-Related Problems

During the preparation for POP surgery in Case 1, routine cervical cytology indicated ASC-H before operation, after which a colposcopy and cervical biopsy were performed. The pathological results indicated high-grade squamous intraepithelial lesions involving the glands, and a further diagnostic LEEP was performed, the results of which indicated stage IA1 cervical squamous cell carcinoma. This suggests that cervical cytology should be performed routinely for all benign gynecological diseases prior to surgical treatment, and the operation should be carried out in strict accordance with the diagnosis and treatment specifications. If a cervical biopsy indicates high-grade lesions or early invasive carcinoma of the cervix that cannot be staged, cervical conization is required, and hysterectomy cannot be immediately performed.

The patient in Case 2 had symptoms of postmenopausal vaginal bleeding and discharge, but did not visit a doctor. An adnexal mass was later found during preoperative preparation for POP surgery. The mass was considered to derive from the fallopian tube, so full preoperative preparation was carried out. Intraoperative frozen-section pathology suggested a malignant fallopian tube tumor. Standardized surgery was performed in time, the correct treatment scheme was selected, and unnecessary secondary surgery was avoided. Primary fallopian tube carcinoma (PFTC) accounts for only 0.14%–1.8% of malignant gynecological tumors (5), is very difficult to cure, and is easily missed or misdiagnosed (6). Although this patient had typical PFTC symptoms, she only sought medical treatment because of the main symptom of POP. This suggests that clinicians should analyze a patient’s condition based on the patient’s medical history, signs, auxiliary examination, and intraoperative exploration, thereby reducing the missed diagnosis and misdiagnosis rate in clinical work.

The patient in Case 3 had no vaginal bleeding or drainage after menopause and visited the hospital for POP. Preoperative ultrasonography revealed that the endometrium was slightly thickened. A hysteroscopy was carried out, the results of which indicated endometrial cancer. A previous study has reported that hysteroscopy can detect canceration and precancerous lesions in only 3% of asymptomatic postmenopausal women with endometrial thickness ≥4 mm (7). It has also been reported that it is reasonable for asymptomatic postmenopausal women with endometrial thickness ≥10 mm to undergo endometrial biopsy or outpatient hysteroscopy and that asymptomatic women with endometrial thickness of 4–10 mm should be further examined according to their specific situation (8). Although the prevalence of endometrial cancer is very low in asymptomatic patients, there is a hidden danger of malignant uterine tumors in patients with POP who retain their uterus. It is suggested that, for patients with POP, especially those who need to preserve their uterus, gynecological color Doppler ultrasound should be performed routinely. Ultrasound is an important examination for gynecological diseases, especially transvaginal ultrasound (TVU) plays a leading role in the diagnosis and treatment of gynecological diseases, and ultrasound has the advantages of economy, safety and non-invasive. It has been reported that TVU has similar sensitivity and specificity to MRI in the treatment of endometrial carcinoma with myometrium invasion and cervical invasion, but with lower cost, better patient tolerance and no need for contrast media (9).

for those with endometrial thickening, a further hysteroscopy is recommended to exclude endometrial lesions.

### The Feasibility of Laparoscopic Total Pelvic Reconstruction Without Mesh in Patients with Malignant Gynecological Tumors and POP

All three patients underwent total pelvic floor reconstruction without mesh during the surgical treatment of malignant gynecological tumors. Severe pelvic organ prolapse have serious impact on patients quality of life, POP combined gynecologic malignant tumor patient in order to guarantee the effect of radiotherapy and reduce the organ prolapse of reset before radiotherapy complications of radiotherapy, radiotherapy after placing pessary improve the symptoms of prolapse, for POP merging of cervical cancer patients, pessary place may oppression tumor, Local infection and bleeding are difficult to control, so it is very necessary to choose a convenient and effective pelvic floor repair operation at the same time in tumor surgery (10).

Study of gynecologic malignant tumor prolapse consolidation at home and abroad is less, adopt the reasonable treatment can treat prolapse and gynecologic malignant tumor at the same time make the treatment of both diseases are to achieve the ideal effect is our purpose, the patients of pelvic organ prolapse surgery implant mesh repair and autologous tissue repair, due to implant rejection, erosion, or secondary infection complications such as serious problem, Especially for gynecological malignant tumors, the operation time is long, the wound surface is large, the risk of infection increases, and the risk of network exposure and erosion increases accordingly. We should pay attention to autologous tissue repair, especially for patients with malignant tumors who need radiotherapy and chemotherapy after surgery. The existence of implants may affect the treatment of the tumor itself. Studies have shown that autologous tissue repair is effective in patients with pelvic organ prolapse in the short and medium term with fewer complications. In addition, patients with gynecological tumor surgery for abdominal or laparoscopic approach, commonly less choice by vaginal surgery, the tumor operation at the same time, choose the self organization pelvic reconstructive surgery in patients with no obvious prolong operation time, increase the complications of the case not only correct the patients with pelvic organ prolapse, and improve the patients quality of life, also can be the greatest degree to reduce the effects of cancer treatment (10). Comprehensive stage malignant tumor by laparoscopic surgery can obviously reduce the operation time, intraoperative hemorrhage, and length of hospital stay, reduce patient pain, prolapse patients laparoscopic autologous fascia revascularization anatomy and surgical field more clearly, hemostatic more thoroughly, a collection of 170 patients with uterine sarcoma of the data comparing laparoscopic surgery and laparotomy, Laparoscopic surgery was found to have more favorable perioperative and postoperative outcomes, with no difference in disease-free survival (DFS) and overall survival (OS) between the two groups (11).

In all cases, the surgical procedure was smooth and without complication, and no abnormalities were found in the short-term, medium-term, and long-term follow-ups.

There are two key issues in pelvic floor reconstruction: top fixation and anterior-to-posterior wall repair. The operation uses the patient’s own tissue to treat POP, which is in line with the principles of restoring anatomy and physiology, preserving function, and minimal invasiveness. The subjective and objective success rate is high. This operation is supported by Delancey’s theory of three chambers and three levels and the double-layer hammock theory. When Lin et al. (12, 13) carried out this procedure, it was found that the recurrence rate was not higher than that of repair with mesh, allowing the side effects associated with the use of mesh to be avoided. The procedure also flexibly adjusts the repair of the anterior-to-posterior walls of the vagina based on the firm fixation of the top according to the different types and degrees of prolapse, emphasizing the integrity of the pelvic floor. If an anterior pelvic prolapse and uterine prolapse are dominant, the high shortening of the uterosacral ligament is used along with the autologous fascia reconstruction of the anterior vaginal wall. If all the anterior, middle, and posterior pelvis has prolapsed, the high shortening of the uterosacral ligament is used along with the autologous fascia reconstruction of both the anterior and posterior vaginal wall. If both the middle and posterior pelvis has prolapsed, the high shortening of the uterosacral ligament is used, along with the autologous fascia reconstruction of the posterior vaginal wall.

This operation is based on laparoscopic visual anatomy, in accordance with the natural space and in line with the basic mechanics of the pelvic floor, so it can more effectively suspend the vagina and maintain its length and sexual function. The use of the patient’s own tissue in the procedure removes the complications associated with mesh and is therefore both economical and practical. The procedure is also relatively simple and has few complications. It can repair and reinforce the horizontal supporting structure of the uterus and vagina, while maintaining the three-chamber structure in the vertical direction of the pelvic floor, resulting in the prolapse recurrence rate not increasing significantly compared with other surgical methods (14). The present study found that, compared with the traditional methods of repairing the anterior-to-posterior vaginal wall, this method has the advantage of finding a reliable anchor point, resulting in the recurrence rate being significantly lower than that of traditional surgery. Previous research has identified surgical complications, including the exposure of sutured ureters, nerves, and vaginal sutures (15). In the cases in the present study, the vesicovaginal space, lateral rectal space, and rectovaginal space were routinely opened and pushed off the ureter and rectum, and the operation was undertaken along the anatomical space, resulting in non-bleeding and non-injury.

Currently, some doctors fail to undertake adequate preoperative preparation in order to shorten patient hospitalization times, but this affects a doctor’s comprehensive and systematic grasp of a patient’s condition (16). A lack of comprehensive analysis of all of a patient’s symptoms, signs, and preoperative examinations can lead to missed diagnosis and misdiagnosis.

## Conclusion

In terms of the treatment of gynecological tumors, patients may face postoperative radiotherapy and chemotherapy, which can affect wound healing. The main complications of mesh are mesh exposure, pain, infection, erosion, micturition, neuromuscular problems, and patient feeling problems (17, 18). Mesh implantation can also increase the short-term or long-term potential risk to patients. If a patient’s POP is not considered during malignant tumor surgery, their quality of life will be affected, so it is vital that comprehensive consideration be given in the selection of treatment options for patients with POP and malignant tumors. Clinicians should not only aim to prolong survival time and improve the prognosis of patients but also improve their quality of life. Laparoscopic total pelvic reconstruction without mesh has few serious complications, and the effects of short-term anatomical reduction and functional rehabilitation are good, making it a suitable surgical method for patients with malignant tumors and prolapse. However, the long-term effects of this procedure require further study.

## Data Availability

The original contributions presented in the study are included in the article/Supplementary Material, further inquiries can be directed to the corresponding author/s.
